# Transplant Tolerance Induction: Insights From the Liver

**DOI:** 10.3389/fimmu.2020.01044

**Published:** 2020-06-05

**Authors:** Helong Dai, Yawen Zheng, Angus W. Thomson, Natasha M. Rogers

**Affiliations:** ^1^Department of Kidney Transplantation, The Second Xiangya Hospital of Central South University, Changsha, China; ^2^Clinical Research Center for Organ Transplantation in Hunan Province, Changsha, China; ^3^Clinical Immunology Center, Central South University, Changsha, China; ^4^Department of Surgery, Thomas E. Starzl Transplantation Institute, University of Pittsburgh School of Medicine, Pittsburgh, PA, United States; ^5^Department of Immunology, University of Pittsburgh School of Medicine, Pittsburgh, PA, United States; ^6^Center for Transplant and Renal Research, Westmead Institute for Medical Research, Westmead, NSW, Australia; ^7^Renal Division, Westmead Hospital, Westmead, NSW, Australia; ^8^Westmead Clinical School, University of Sydney, Westmead, NSW, Australia

**Keywords:** liver transplantation, immune tolerance, mechanisms, cell therapy, immunosuppression withdrawal

## Abstract

A comparison of pre-clinical transplant models and of solid organs transplanted in routine clinical practice demonstrates that the liver is most amenable to the development of immunological tolerance. This phenomenon arises in the absence of stringent conditioning regimens that accompany published tolerizing protocols for other organs, particularly the kidney. The unique immunologic properties of the liver have assisted our understanding of the alloimmune response and how it can be manipulated to improve graft function and survival. This review will address important findings following liver transplantation in both animals and humans, and how these have driven the understanding and development of therapeutic immunosuppressive options. We will discuss the liver's unique system of immune and non-immune cells that regulate immunity, yet maintain effective responses to pathogens, as well as mechanisms of liver transplant tolerance in pre-clinical models and humans, including current immunosuppressive drug withdrawal trials and biomarkers of tolerance. In addition, we will address innovative therapeutic strategies, including mesenchymal stem cell, regulatory T cell, and regulatory dendritic cell therapy to promote liver allograft tolerance or minimization of immunosuppression in the clinic.

## Introduction

The location and anatomy of the liver, positioned between the gastrointestinal tract and the systemic circulation, allows it to conduct its functions of digestion, synthesis of plasma proteins and detoxification (1). Circulating blood from the gastrointestinal tract enriched with food antigens (Ags) and environmental microbial products, including endotoxin, converge in the liver portal vein (2). The hepatic artery, which provides about 20% of the liver blood supply, and the hepatic portal vein mix in the liver to create sinusoids. Liver sinusoidal endothelial cells (LSEC) are located in the space of Dissé and form an immediate barrier between the hepatocytes and the bloodstream (1, 3). The non-parenchymal cell populations including dendritic cells (DC), Kupffer cells (KC), and LSEC constitute the hepatic reticulo-endothelial system, which is responsible for clearing Ags and degradation of toxins from sinusoidal blood by uptake through endocytic receptors (1). The cross-talk between T cells and liver parenchymal cells, including LSEC, hepatocytes, hepatic stellate cells, and cholangiocytes, plays a crucial role in tolerance induction (4).

“Spontaneous” liver transplant tolerance has been demonstrated in both animals and humans, however, the mechanisms that underlie development of tolerance to the liver but not to other solid organ grafts are still not well-understood. We will summarize recent research findings, focusing on (i) the specific contributions of immune cells, mesenchymal stem cells (MSC) and parenchymal cell subsets that promote a tolerogenic microenvironment within the liver, (ii) mechanisms of organ-specific tolerance, and (iii) novel strategies to predict and promote liver transplant tolerance.

## Intrahepatic Immune Cells Interact with Liver Parenchymal Cells to Generate a Tolerogenic Microenvironment

Unlike conventional DC in secondary lymphoid tissue, both mouse and human liver DC display tolerogenic properties (5–8). Liver DC express comparatively low levels of Toll-like receptor 4 (TLR4) that limits their response to specific ligands, leading to reduced hepatic adaptive immune response (8). Similarly, freshly-isolated, unmanipulated murine liver non-conventional plasmacytoid DC (pDC) express low levels of co-stimulatory molecules and weakly stimulate T cell responses (9, 10). Liver pDC prevent oral T cell priming through inducing anergy or deletion of circulating T cells via a CD4^+^ T cell-independent mechanism (11). Monocytes cultured with hepatocyte growth factor or liver epithelial cells can differentiate into DC that release high levels of IL-10 (12, 13), suggesting that the hepatic microenvironment modulates DC differentiation into regulatory subsets (14).

KC located in the hepatic sinusoids are recognized as tissue-resident macrophages, originally derived from the blood monocytes (2). KC can phagocytose apoptotic cells and microorganisms, and therefore function similarly to other organ-based macrophages (2, 15). KC are also involved in portal venous tolerance, where Ag administration into the portal vein induces specific tolerance to that Ag. The mechanism for this type of tolerance appears to be KC-based release of IFN-γ-stimulated nitric oxide (NO) that inhibits T cell proliferation (16). KC treated with gadolinium chloride prevented the induction of portal venous tolerance by inhibiting Ag presentation to lymphocytes, supporting the notion that both Ag presentation to and stimulation of lymphocyte proliferation are necessary for tolerance induction (17). In human studies, a greater number of KC typically found in younger living donors predicts better hepatic allograft survival compared to elderly living donors, suggesting that KC in the donor liver are a relevant prognostic factor influencing post-transplant outcomes (18). Graft- infiltrating DC and KC were also shown to be increased during and after rat liver transplant tolerance induction, again suggesting a possible important role for these cells in shaping the host immune response toward tolerance (19).

Mouse LSEC express the mannose receptor and the scavenger receptor to enhance Ag uptake, and also express co-stimulatory molecules, including CD40, CD80, and CD86 that facilitate Ag presentation and T cell stimulatory function (20). Human LSEC constitutively express CD40, but CD80/CD86 is inducible and expressed during inflammation (21). Therefore, murine and human LSECs might function differently. Mouse LSEC can present circulating exogenous Ags to CD4^+^ T and CD8^+^ T cells, resulting in Ag-specific T cell tolerance, but not Th1 responses (22, 23). LSEC synthetic and endocytic function has been shown to be greater in spontaneously tolerant rat liver allografts compared to those that were rejected (24). LSEC lectin uniquely recognizes activated T cells and negatively regulates their responses (25). In addition, the threshold of Ag expression within the liver is the dominant factor determining T cell fate, rather than Ag cross-presentation, since low-level hepatocyte expression of cognate Ag generates an effector response that becomes functionally silenced at a high level of the same Ag (26).

Regarding lymphocytes, the hepatic CD8^+^: CD4^+^ T cell ratio is higher compared to peripheral blood (27), and both natural killer (NK) and natural killer T (NKT) cells are present at a higher percentage (of total cells) compared to that in secondary lymphoid organs. In contrast to T cells activated by splenocytes, T cells activated by hepatocytes lose cytolytic function after 3 days of co-culture and fail to survive (28). The mechanism of hepatocyte-induced T cell death is neither Fas (CD95)- nor tumor necrosis factor (TNF) receptor-dependent, suggesting a type of apoptosis known as passive cell death (29). In both murine and human liver transplantation, T cell infiltration into allografts is followed by their apoptosis (30, 31). Mouse liver CD8^+^ T cells are also programmed to die following intrahepatic activation in a pro-apoptotic protein Bim-dependent manner (32). However, the molecular recognition events that induce apoptosis of graft-infiltrating T cells, and the reason why this phenomenon occurs within the liver, but not other allografts is unclear (30, 33).

Mesenchymal stem (stromal) cells (MSC) display unique immunosuppressive and anti-inflammatory properties that may modulate allograft outcomes. Adult liver-derived MSC are negative for human leukocyte Ag class II (HLA-II) and the co-stimulatory molecules, including CD80 and CD86, which can inhibit the proliferation of T cells activated by mitogen (34). Interestingly, liver graft-derived MSC have greater capacity to suppress allo-reactive T cell proliferation and cytotoxic degranulation than bone marrow-derived MSC (BM-MSC) (35), as well as significantly higher levels of immune-regulatory genes than adipose tissue-derived MSC and BM-MSC, that depend on programmed cell death ligand 1(PDL1) expression (36) for their ability to subvert T cell response.

## Comparing the Intrinsic Tolerogenicity of the Liver Grafts in Animals and Humans

In the first report showing spontaneous tolerance induction by liver transplantation, pig hepatic allografts demonstrated long-term survival without immunosuppression, protecting other donor-specific tissue but not third-party organs from rejection (37). This phenomenon was subsequently replicated in pre-sensitized rats that failed to reject donor liver grafts, inducing Ag-specific tolerance in 50% of recipients (38). To avoid the toxicities of irradiation in a sick liver failure recipient, delayed tolerance induction has been promoted when the recipients have recovered post-operatively. An ACI-to-Lewis rat (allogeneic) liver transplant model developed chronic rejection, however, in the same strain combination, liver recipients receiving 100 × 10^6^ T cell-depleted donor BM cells at 3–4 weeks post-transplant followed by tacrolimus withdrawal became tolerant. Mechanistically, this delayed tolerance induction is associated with increased mixed chimerism, Treg generation, and decreased donor-specific antibody (DSA). However, the authors did not investigate key mechanisms underlying the development of delayed tolerance (39). Allogeneic liver transplantation from DA-to-Lewis rats receiving post-transplant total lymphoid irradiation, which is a non-myeloablative regimen to induce graft-infiltrating T cell apoptosis and subsequent accumulation of Treg, also induced tolerance (40). The micro RNA (miRNA) profile in these tolerant allografts was similar to syngeneic grafts, indicating that tolerance potentially returned recipients to a state of immunological quiescence (40). Tolerance to liver transplants in rats can subsequently induce tolerance to intestinal allografts by hampering the expression of IL-2 receptor on recipient CD8αβ^+^ lymphocytes in the lamina propria and reducing recruitment of NK cell and macrophages (41).

Spontaneous liver transplant tolerance between MHC-disparate murine strain combinations is significantly higher than that seen with kidney or heart allografts (42–44), and is summarized in Table 1. In the murine orthotopic liver transplantation model (68), allografts were accepted in 13 mouse strain combinations that showed evidence of donor cell chimerism (42). Mouse liver allografts can rescue donor-specific skin transplants from rejection, either pre- or post-liver transplant (42).

**Table 1:** A comparison of the intrinsic tolerogenicity of liver grafts with other transplanted organs in animals and humans.

**Species**	**Donor/recipient**	**Graft survival time**						**References**
		**Liver**	**Kidney**	**Heart**	**Skin**	**Co-D-Skin**	**Co-T-Skin**	
Mouse	C57BL/6 → BALB/c	70% > 100 d	39.3 ± 3.1 d	8.3 ± 1.6	<10 d	80% > 100 d	18 ± 5 d	(42, 44–48)
	BALB/c → CBA	57% > 100 d	7.5 ± 1.5 d	8.6 ± 0.9	8.5 ± 1.5 d	/	/	
	C57BL/6 → C3H/HeN	73% > 100 d	7.5 ± 1.5 d	8.1 ± 0.8	10.6 ± 0.9	/	/	
Rat	DA → PVG	80% > 100 d	12 d	8 d	6 ± 2 d	75% > 100 d	8 ± 1	(38, 49–52)
Pig	Landrace → Landrace	>18 month	7 d	6.5 ± 1.5d	9 ± 3 d	>24d	11 ± 4 d	(37, 53, 54)
NHPs	Cynomolgus monkeys → cynomolgus monkeys	<7 months	<2 wks	<2 wks	6 ± 1 d	/	/	(55–58)
Human		Liver allograft achieved “operational tolerance”						(59–63)
		Advantage of liver co-transplant: protection to kidney and heart grafts						(64–67)

*Co-D-Skin, Co-transplant donor derived skin with liver; Co-T-Skin, Co-transplant third-party skin with liver; d, days; NHPs, non-human primates; wks, weeks*.

Human liver allograft “operational tolerance” has also been described and reviewed by many investigators (59–63, 69). Hepatic allografts protect simultaneously transplanted kidney allografts from the same donor from chronic cell- and antibody-mediated immune injury, resulting in better graft function compared with kidney transplant alone (64, 65). Combined liver and heart transplantation shows less evidence of cardiac allograft vasculopathy than isolated heart transplantation when detected by coronary three-dimensional volumetric intravascular ultrasound (66). Simultaneous liver-heart transplantation also showed reduced T cell-mediated rejection compared with cardiac transplantation alone (67).

The question of why only the liver displays inherent tolerogenicity is worthy of consideration. The naïve mouse liver has a greater number of DC than other parenchymal organs, such as heart, kidney, and pancreas (70). Recent findings reveal that DBA2J pDC are more powerful in inducing forkhead box p3 (Foxp3) expression in C57BL6 T cells and promoting kidney graft tolerance than the reverse combination. This suggests that the organ- and strain-specific differences exist that determines tolerance (71). In human studies, donor-reactive T cell clones were reduced in three tolerant combined kidney and BM transplant recipients, but not in non-tolerant patients (72). However, the same group further reported that donor-reactive T cell clone reduction was not associated with liver graft tolerance or failure, again highlighting organ-specific tolerance mechanisms in patients (73).

## Understanding the Mechanisms of Experimental Liver Transplant Tolerance

The literature on mechanisms that underlie liver allograft acceptance in rodent models is extensive, but centers on immunoregulation, and an intrinsic balance between leukocyte, non-parenchymal-parenchymal cell ratios, effector, and regulatory T cells, Ag-presenting cell phenotype, and function, as well as cross-talk between cellular compartments. The identification of molecular pathways that alter immunoregulation provides promising potential therapeutic avenues for clinical application. Liver transplant acceptance is also characterized by donor-specific hypo-responsiveness, mimicking the tolerance arising from chimerism following BM transplantation. The development of liver allograft tolerance appears to be independent on the thymus (74, 75). Current experimental results favor deletion of alloreactive T cells occurring within the organ and secondary lymphoid tissue, leading to a reduced burden of effector cells. Hepatic DC differ in their maturation state and allostimulatory capacity compared to DC isolated from other solid organs (76), and their capacity to modulate T cell function is well-known. However, the relative contribution of innate immune subsets like DC and NK cells has not been characterized.

**Figure 1:**
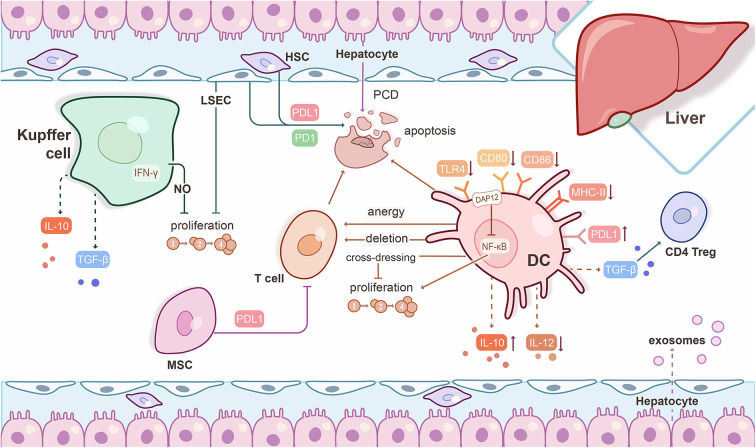
Mechanisms underlying experimental liver transplant tolerance. Hepatic immune and parenchymal cells interact with each other to generate a tolerogenic microenvironment. Liver dendritic cells (DC) express low levels of Toll-like receptor 4 (TLR4) and co-stimulatory molecules, but high levels of PDL1, weakly stimulate T cell responses, and promote regulatory CD4^+^ T cells (CD4 Treg) induction through TGF-β. Liver DC release high levels of IL-10, but low bioactive IL-12. Liver DC prevent T cell priming of orally-administered Ag through anergy or deletion of circulating T cells. Graft-infiltrating, cross-dressed DC over-express PDL1 and subvert anti-donor T cell proliferation to promote liver graft tolerance. The DNAX-activating protein of 12 kDa (DAP12) negatively regulates liver DC IL-12 production, but positively regulates liver DC IL-10 production and T cell allostimulatory capability. Kupffer cells can release IFN-γ-stimulated nitric oxide (NO) to inhibit T cell proliferation and produce IL-10 and TGF-β to promote tolerance. Liver sinusoidal endothelial cells (LSEC) present circulating exogenous antigens to T cells, resulting in Ag-specific T cell tolerance. LSEC and hepatic stellate cells (HSC) induce T cell apoptosis through PDL1/PD1 pathway interactions. The mechanism of hepatocyte-induced T cell death occurs through a type of apoptosis known as passive cell death (PCD). Exosomes derived from hepatocytes may also be critical to a tolerogenic phenotype. Mesenchymal stromal cells (MSC) suppress T cell proliferation and differentiation through cell-cell contact that is mediated by PDL1.

Regardless of strain combination in rodent liver transplantation models, spontaneous tolerance appears to be induced by the graft itself, with liver-derived cell populations silencing the host immune response (77) (Figure 1). This feature is strain- and organ-specific: Lewis rat liver allografts demonstrate prolonged survival in DA recipients, although the reverse combination results in acute rejection. Rejection has been characterized by hepatocyte death, but allograft acceptance is associated with apoptotic mononuclear cells and upregulated FasL parenchymal expression. Irradiated Lewis rat donor livers lost tolerogenic capacity highlighting the role of hepatic passenger leukocytes (77). Donor passenger leukocytes, particularly T cells, but not B cells and macrophages, prolong irradiated donor liver allograft survival in the PVG-to-DA combinations, but reject transplanted heart grafts (78). Adoptive transfer of donor leukocytes or splenocytes re-establishes recipient tolerance, but not following T cell depletion. Interestingly, when two kidneys and two hearts of PVG rats were transplanted into each DA recipient, along with adoptive transfer of high dose donor leukocytes (1.5 × 10^8^), transplanted organs were accepted, suggesting that liver-derived spontaneous transplant tolerance may be determined by the ratio of donor leukocytes to the quantity of donor tissues (78). Donor passenger leukocytes from transplanted liver grafts migrate rapidly into recipient lymphoid tissues, but their numbers decrease dramatically within the first 48 h (79), accompanied by deletion of alloreactive CD8^+^ T cells. Higher levels of apoptosis of infiltrating leukocytes within liver allografts are seen compared to renal allografts in the same rat strain combination (PVG-to-DA) (80). T cell clonal deletion (81, 82) was initially proposed as the cause of liver allograft acceptance. However, lymphocytes from long-term survival recipients demonstrate vigorous Ag-specific responses *in vitro* (83). Donor liver leukocyte-induced recipient T cell death by neglect also appears to be responsible for liver acceptance (77, 84). Deletion of donor passenger leukocytes by irradiation of the donor rat followed by liver transplantation breaks allograft acceptance (85). However, other studies have failed to confirm that the presence of donor passenger leukocytes is associated with allograft tolerance (86).

T cell apoptosis in the liver graft plays a crucial role in tolerance. Interferon (IFN)-γ is a key inflammatory cytokine produced by effector T cells. Surprisingly, IFN-γ knockout liver allografts are acutely rejected (87), suggesting that intact signaling is necessary for graft tolerance. T cell-derived IFN-γ signaling results in hepatic stellate cell and LSEC expression of PDL1, inducing T cell apoptosis through the PDL1/PD1 pathway (88). Functional assessment of these cells isolated from tolerated liver grafts demonstrated inhibition of T cell proliferative responses, particularly those of CD8^+^ T cells. These findings were replicated in human CD45^−^ non-parenchymal cells that limited peripheral blood mononuclear cell (PBMC)-derived T cell proliferation. Blocking this pathway using anti-PDL1 antibody (Ab) or using PDL1 knockout mice as donors resulted in allograft rejection, highlighting the essential role of PDL1 expression in the liver parenchyma to regulate apoptosis of alloreactive cells (89). Cytotoxic T-lymphocyte-associated protein 4 (CTLA4) blockade prevents T cell apoptosis and induces acute rejection, suggesting such signaling is also a pre-requisite for spontaneous mouse liver transplant tolerance (90). Anti-CTLA4 treatment enhances NK cell cytotoxicity, and augments IL-2 and IFN-γ in both graft and recipient spleen, in keeping with lack of alloreactive T cell death. Galectin-1, an endogenous lectin expressed in lymphoid organs, is upregulated in liver allografts and administration of recombinant protein significantly prolongs liver allografts. This was associated with enhanced CD4^+^ and CD8^+^ T cell apoptosis in the graft itself and recipient spleen and suppression of Th1/Th17 cell responses. There was no suggestion of modulation of regulatory effects by altering CD4^+^CD25^+^FoxP3^+^ T cell numbers (91). Overexpression of galectin-1 in T cells promotes the activation of hepatic stellate cells that contribute to tolerance (92).

Regulatory T cells (CD4^+^CD25^+^FoxP3^+^ Treg) have been demonstrated to increase significantly in the recipient liver graft and spleen. Moreover, depletion of recipient CD4^+^CD25^+^ T cells using anti-CD25 (IL-2Rα) Ab reduces apoptosis of graft-infiltrating CD4^+^ and CD8^+^ T cells, leading ultimately to liver allograft rejection (93). These findings highlight the roles of both CD4^+^ Tregs (94, 95) and apoptosis of graft-infiltrating T cells in liver transplant tolerance induction. The CD8^+^CD103^+^ T cell subset possess suppressive function and also contributes to spontaneous liver graft tolerance, but the specific mechanism of action remains unclear (96). IFN-γ deficient liver allografts that reject around day 15 post-transplant show similar levels of Tregs but less T cell apoptosis compared to wild-type allografts, suggesting that T cell elimination may be the more critical factor (88). These data are further supported by observations in a B10-to-C3H mouse liver transplant model which showed that T cell deletion, not regulation, was responsible for spontaneous graft acceptance (30).

The role of NK cells in organ transplantation is still controversial (97–100). NK cells have been identified as a potential predictor of liver transplant tolerance (101). There are multiple potential mechanisms of action including direct lysis of recipient CD4^+^ and CD8^+^ T cells (102), deletion of Ag-presenting cells (103), and CD8^+^ T cell hypo-responsiveness (104) which have been summarized elsewhere (99). However, NK cells in rat liver allografts can also promote rejection by producing IFN-γ in the early post-transplant period (105).

Host DC acquire donor major histocompatibility complex (MHC) molecules after mouse orthotopic liver transplant, to appear as “cross-dressed” DC (CD-DC). Graft-infiltrating CD-DC expressed PDL1 and IL-10 that subvert anti-donor T cell responses and promote death of graft-infiltrating CD8^+^ T cells to promote liver graft tolerance (106). The transmembrane immuno-adaptor DNAX-activating protein of 12 kDa (DAP12) has been shown to negatively regulate conventional liver myeloid DC maturation, migration to host lymphoid tissue, and T cell allo-stimulatory capability (107, 108). DAP12^−/−^ liver grafts exhibit low levels of Tregs and fail to induce liver transplant tolerance (107).

The balance of pro- and anti-inflammatory cytokines as well as other molecules within the hepatic microenvironment can crucially influence adaptive immune responses. Intrahepatic IL-4 transcripts were significantly lower in tolerated rat liver allografts compared to rejected allografts, however, no significant differences were observed for other cytokines (including IL-1α, IL-2, IL-6, IL-10, TNF-α, TNF-β, and transforming growth factor β (TGF-β) (109). IL-4 injection after rat liver transplantation converts allograft tolerance to rejection partially through a graft-specific antibody response (110). In the murine tolerant liver allograft, expression of miRNA-146a, 15b, 223, 23a, 27a, 34a, and 451 is upregulated compared to syngeneic grafts, suggesting a role for miRNA in tolerance induction (111). Expression of lectin galactose-binding soluble 1, fibrinogen-like protein 2 (Fgl2), the ectoenzyme CD39, phosphodiesterase 3B, killer cell lectin-like receptor G1 (Klrg1), Foxp3, and TGF-β, have all been shown to increase at 8–14 days following murine liver transplantation and promote tolerance to the allografts (112). However, the cellular origins of these factors are non-specific and may represent a combined signal from hepatocytes, infiltrating leukocytes, and non-parenchymal cells. The use of cutting-edge single-cell sequencing techniques will allow us to improve on these preliminary findings.

## Monitoring and Prediction of Clinical Liver Transplant Tolerance

Development of non-invasive biomarkers as diagnostic tools to define graft tolerance remains an important area of research in liver transplantation (113). Reliable, non-invasive biomarkers to predict graft rejection are not currently available, but are urgently needed (63). A prospective, longitudinal, international multi-center cohort study on immune monitoring after pediatric liver transplant is ongoing (114), and will provide much-needed data discovery and validation.

In order to investigate immunologic mechanisms elicited by immunosuppression (IS) withdrawal, 24 operationally tolerant recipients and 14 non-tolerant recipients were selected for analysis of T cell subset infiltration and gene expression pattern in protocol liver biopsy specimens prior to weaning, as well as 1 and 3 years after IS withdrawal. Treg reduction to baseline levels in liver biopsies, in addition to down-regulation of immune activation-associated genes at 3 years post-withdrawal in the context of no graft damage, suggested a balanced immune response in tolerant recipients (115). The dynamic profile of Treg in liver transplant recipients during IS weaning was explored by monitoring the frequency of Treg and Foxp3 mRNA expression in PBMC in 12 liver transplant patients undergoing IS withdrawal. A progressive increase in circulating CD4^+^CD25^+^Foxp3^+^ Treg and Foxp3 mRNA expression was associated with operational tolerance in liver transplant recipients (14, 116). The expression of adenosine deaminase, which degrades adenosine to evoke stronger Treg activation, was higher in five tolerant liver transplant patients compared to the 12 non-tolerant recipients. These data indirectly indicate that adenosine deaminase potentially predicts liver transplant tolerance through targeting Treg (117). Using single-cell mass cytometry to detect immune profiles in peripheral blood of seven operational tolerant pediatric recipients and eight pediatric recipients on low dose single agent IS, a specific CD4^+^ T cell subset that is CD4^+^CD5^+^CD25^+^CD38^−/low^CD45RA^−^, distinct from Treg, correlated with liver allograft tolerance. This specific T cell subset lacks both CD45RA and stable Foxp3 expression, but expresses CD5 that has been shown to be crucial in promoting Treg induction (118).

Immune cell ratios and their balance can predict tolerance vs. rejection. A comparison of 19 liver transplant patients on IS, operationally tolerant liver transplant recipients or 24 age-matched healthy volunteers demonstrated an increased frequency of CD4^+^CD25^+^ T cells and B cells, altered Vδ1/Vδ2γδ T cell ratio, but decreased NK cells in PBMC in operationally tolerant patients (119). The ratios of Treg/Th17, Th1/Th17, and CD8^+^/Th17 cells were increased in tolerant patients compared with non-tolerant patients during immunosuppression tapering. The elevated Treg/Th17 ratio continued over 60 months follow-up in tolerant patients, indicating a reciprocal balance between Treg and Th17 that may contribute to the development and maintenance of tolerance (120). Tolerant liver recipients also exhibit greater numbers of CD4^+^CD25^+^ T cells and Vδ1^+^ T cells in the circulation compared to non-tolerant patients and healthy individuals (121). Adult liver allografts also contain a small population of hematopoietic stem/progenitor cells (Lin^−^CD34^+^CD38^−^CD90^+^) that may promote long-term (6 months to 8 years) chimerism in the graft (122). The ratio of DC precursors CD11c^−^CD123^hi^ (pDC2) to CD11c^+^CD123^−/low^ (pDC1) was also significantly higher in 36 patients undergoing successful drug weaning compared to those 21 patients on maintenance immunosuppression, regardless of the dose of prednisone or tacrolimus. These data suggest that pDC2 that can polarize naïve Th cell toward a Th2 phenotype may drive tolerance induction (123). In a further study, 13 tolerant liver transplant recipients showed an elevated ratio of plasmacytoid DC (pDC) to myeloid DC compared to those 12 patients remaining on immunosuppression. Additionally, a high PDL1/CD86 ratio on pDC correlated with increased Treg and correlated with pediatric liver allograft tolerance (124).

Gene expression of sentrin-specific peptidase 6 (SENP6) and Fem-1 homolog C (FEM1C) were shown to be predictive biomarkers of liver transplant tolerance in a single cohort of 17 liver transplant recipients (125). At least 13 unique gene sets, including SENP6 that is associated with NK cells, were significantly expressed in adult and pediatric liver transplant patients, which showed a prediction for tolerance (126). This conclusion was supported by previous findings of differential gene expression between tolerant and non-tolerant transplant recipients within the NK cell compartment despite no clear differences in absolute cell number between these patient groups (101). The intra-liver allograft gene expression involved in the regulation of iron homeostasis is more active in operationally tolerant patients compared to non-tolerant recipients and independent of baseline immunosuppression (127). However, the iron-related markers were poor predictors for drug withdrawal in hepatitis C virus (HCV)-infected liver transplant recipients (128), which could be due to inhibition of hepcidin expression by HCV (129). Regardless, the blood gene expression was not sensitive enough to distinguish rejection vs. HCV-infection (130). However, type I IFN-stimulated gene overexpression within liver allografts of HCV-positive recipients, along with circulating PD1/CTLA4/2B4-positive HCV-specific CD8^+^ exhausted T cells, were associated with liver graft operational tolerance induction (128).

Single-cell RNA sequencing (scRNAseq) can provide a comprehensive map to characterize human hepatic immune cell populations and also non-parenchymal cells (131), and it is anticipated that it may prove helpful in predicting liver transplant rejection vs. tolerance capacity in the near future. However, before validated accurate, non-invasive biomarkers are available, histopathological findings remain the gold standard to determine the management of immunosuppression (132).

## Ongoing and Novel Therapeutic Approaches to Promote Liver Transplant Tolerance in Patients

Life-long immunosuppression and its accompanying burden of increased morbidity and mortality has prompted interest in immunosuppressive drug withdrawal (133). In the first multi-center trial of drug withdrawal in adult liver transplant recipients, 41.84% of evaluated recipients were successfully weaned from immunosuppression at least 3 years post-transplantation (134). In the first multi-center immunosuppression withdrawal trial in pediatric recipients of parental living donor liver transplantation, complete cessation of immunosuppressive agents for at least 1 year showed normal graft function and stable liver graft biopsies (60). The majority of these promising clinical trials have been documented in detail elsewhere (132, 135).

Several factors could potentially affect the outcomes of drug withdrawal. The interval between transplantation and initiation of drug withdrawal appears to be one of the most powerful clinical predictors of success (136, 137), as a longer post-transplant period (131 ± 43 vs. 83 ± 40 months) may establish better host-graft adaptation (134). Over 60% of liver transplant recipients with a longer time interval (156 months post-transplant) and a lower lymphocyte proliferation index became clinically tolerant at a median of 14 months of follow-up (138). Younger recipients at the time of transplantation had better outcomes and a higher possibility of successful weaning compared to older recipients (139, 140), suggesting that an “adapted” or “inexperienced” immune system was important in drug withdrawal (141). Immunosuppression, including high-dose antithymocyte globulin (ATG) induction followed by short-term rapamycin withdrawal at an early time-point (4 month post-transplant) failed to induce operational liver transplant tolerance, which was associated with CD8^+^ memory T cell expansion and elevated IL-17^+^ cell infiltration in liver grafts (142). Moreover, fewer donor-recipient human leukocyte antigen (HLA)-A-, B-, and DR-mismatches, and a lower incidence of early rejection were associated with successful drug withdrawal in a 3 year follow-up of 18 liver transplant recipients (143).

Due to immunosuppressive drug non-specificity, drug toxicity, inconsistent outcomes, and the difficulty of early complete immunosuppression withdrawal, other strategies, including the use of stem cells, regulatory dendritic cells (DCreg) and Treg therapy have emerged to promote liver allograft tolerance (144–148). Published trials are summarized in Table 2. The pivotal role of many of these cellular subsets in immunomodulation makes them ideal candidates for use as therapeutic agents. Mesenchymal stem cells have the advantage of being sourced from diverse tissues, but they lack a definitive marker to enable isolation. They display low immunogenicity and have been shown to modulate other immune and non-parenchymal cells (157, 158). Immature or regulatory DC have a well-established capacity to induce Ag-specific hypo-responsiveness, Th1 cell apoptosis, and Treg development. Indeed, this phenotype may be enhanced in hepatic DC (6). Treg have the capacity to migrate to sites of inflammation and exert immunosuppressive effects on CD4^+^ and CD8^+^ T cells directly or through elaboration of inhibitory cytokine production. Several studies have reported increased frequency of Tregs in operationally tolerant liver transplant recipients (121) and following weaning of immunosuppression (116). Chimeric Ag receptor or CRISPR/Cas9 technology has recently been applied to modify Treg to enhance their regulatory function *in vitro* (159, 160), and their safety and longevity *in vivo* (161).

**Table 2:** Strategies to promote liver transplant tolerance using cell therapy in the clinic.

**Cell type:** **Authors**	**Phase**	**NCT number**	Date^*^	**Donor**	**Number of** **Patients**	**Infusion** **time**	**Cell** **dose(s)**	**Cell** **source**	**Outcomes/status**	**References^&^**
**MSC**										
Popp et al. (149)	I	NCT01841632	Nov. 2011	DD	3–24	POD 1 and 3	2 doses, 300 × 10^6^	Third Party BM-MAPC	The study objective is to evaluate the safety and clinical feasibility	(149)
Detry etal. (150)	I–II	NCT01429038	Mar. 2012	DD	10	POD 3 ± 2	3 doses, 1.5–3 × 10^6^/kg BW	Third Party MSC	No side effect of infusion. Tolerance was not observed	(150)
Zhang etal. (151)	I	NCT02223897	Jan. 2013	&	12 with ITBL	Weeks 1, 2, 4, 8, 12, 16 after recruitment	6 doses, 1 × 10^6^/kg BW	UC-MSC	No MSC-related side effects. Better graft survival than the control group	(151)
Qi Zhang et al.	I–II	NCT01844063	Jul. 2013	&	210 with graft failure	&	&	UC-MSC	Recruiting	&
Yang et al.	I–II	NCT02706132	Feb. 2014	&	15	&	6 doses, 1 × 10^6^/kg BW	MSC	Recruiting	&
Lorini et al.	I	NCT02260375	Oct. 2014	&	20	&	1 dose, 1–2 × 10^6^/kg BW	Third Party BM derived MSC	Recruiting	&
Soeder et al. (152)	I	NCT01841632	Jun. 2015	Living	1	POD 0 and 2	2 doses, 300 × 10^6^	MAPC	No acute complications with cell infusion. Normal liver function.	(152)
Rutgers et al.	I	NCT02557724	Sep. 2015	Living	&	&	&	&	Recruitment completed	&
Sturm et al.	I	NCT02957552	Mar. 2017	Living	7	POD 0 and 2	2 doses, 1 × 10^6^/kg BW	Donor BM-MSC	Recruiting	(153)
Shi et al. (154)	I–II	NCT01690247	Sep. 2017	DD	13 with ACR	Rejection time	1 dose, 1 × 10^6^/kg BW	UC-MSC	No side effects. ALT decreased with increased Treg/Th17 ratio in the grafts compared with no infusion control	(154)
**Treg**										
Todo et al. (155)	I–II	UMIN000015789	Nov. 2010	Living	10	POD 13	1 dose, 0.23–6.37 × 10^6^/kg BW	Donor Lymphocytes	No side effects; Normal graft function in all patients. Seven patients withdrew IS and three patients developed ACR during weaning IS. No control group data.	(155)
Lombardi et al.	I–II	NCT02129881	May. 2014	Living	15	POD 5	1 dose, 1 × 10^6^/kg BW	Host blood derived Treg	Recruitment completed	&
Feng et al.	I	NCT02188719	Dec. 2014	&	15	&	&	darTregs	Terminated	&
Lu et al. (96)	I	NCT01624077	Dec. 2014	Living	1	POD 0–2 years	1 × 10^6^/kg BW	Host blood derived Treg	Active, not recruiting	&
Feng et al.	II–III	NCT02474199	Jun. 2016	Living	14	POM 24–84	300-500 x10^6^/kg BW	Host blood derived Treg	Recruitment completed	&
Curry et al.	II	NCT02739412	Nov. 2016	&	7	&	&	&	Active, not recruiting	&
Sanchez-Fueyo et al. (156)	IV	NCT02949492	Dec. 2017	&	6	POY 2–6	&	&	Terminated	&
Sanchez-Fueyo, et al. (156)	I	NCT02166177	Nov. 2019	DBD/DCD	9	POM 3–16	1–4.5 × 10^6^/kg BW	Host blood polyclonal Treg	Infusion is safe. Increased circulating Tregs and reduced allo-reactive T cell response	(156)
**DCreg**										
Thomson et al.	I–II	NCT03164265	Aug. 2017	Living	15	POD-7	1 dose, 2.5–10 × 10^6^/kg BW	Donor blood monocyte-derived DCreg	Enrolling	&

*ACR, acute cellular rejection; BM, bone marrow; BM-MAPC, bone marrow-derived-multipotent adult progenitor cells; BM-MSC, bone marrow-derived mesenchymal stem cells; BW, body weight; darTregs, donor-alloAg-reactive regulatory T cells; DBD, donation after brain death; DCD, donation after circulatory death; DCreg, regulatory dendritic cells; DD, deceased donation; IS, immunosuppression; ITBL, ischemic-type biliary lesions; Kg, Kilogram; M, million; MAPC, multipotent adult progenitor cells; MSC, mesenchymal stem cell; N, number; NCT, National Clinical Trial; NIAID, National Institute of Allergy and Infectious Diseases; POD, post-operative days. POM, post-operative months; POY, post-operative years; Ref, reference; UC-MSC, umbilical cord derived mesenchymal stem cell*.

*&means data not available or unpublished*.

* *Date means the trials released date or patient enrolled date or paper published date as reference indicated*.

& *Unpublished data come from ClinicalTrials.gov (https://clinicaltrials.gov/ct2/home) or UMIN Clinical Trials Registry (http://www.umin.ac.jp/ctr/index.htm)*.

A phase I–II study enrolled 10 liver transplant recipients who received 1.5–3 × 10^6^/kg third-party MSC on post-operative day 3 ± 2, and were compared with 10 liver transplants without MSC. This study demonstrated safety, but did not promote tolerance (150). A phase I study of MSC in liver transplantation showed that two infusions of 1.5 × 10^8^ third-party, multi-potent adult progenitor cells into a living-related liver transplant recipient at day 0 and 2 post-transplant was feasible and safe. However, no further follow-up data was reported (152). An open-label, prospective pilot trial of two intravenous infusions of 1 × 10^6^ cells/kg of donor-derived MSC in pediatric living-donor transplant recipients who will receive standard immunosuppression is currently ongoing (153).

A first-in-human clinical trial of donor-derived DCreg infusion to achieve early complete immunosuppression withdrawal and potentially tolerance induction in living donor liver transplant patients is ongoing at the University of Pittsburgh (146, 162, 163) and shows no side effects of cell infusion (published as an abstract in the American Journal of Transplantation 2019). Five registered clinical trials of Treg cell therapy have previously been detailed (144) and are summarized in Table 2. Infusion of *ex vivo*-generated host-derived donor Ag alloreactive Tregs into 10 consecutive adult recipients early post-liver transplant following cyclophosphamide showed safety and efficacy for immunosuppression withdrawal and clinical tolerance induction in 7 out of 10 patients (155).

*In vitro* study shows that targeting primary human hepatocytes by silencing their HLA class I expression can alleviate alloreactive T cell proliferation without impairing metabolic function (164). In contrast to this human finding, adeno-associated viral vector transfer of donor MHC-I molecule to recipient hepatocytes can induce allospecific CD8^+^ Treg expansion, and promote allogeneic pancreatic islet graft tolerance (165). However, targeting of HLA expression is currently far from progressing to clinical practice.

## Conclusions

The liver, an atypical immune and metabolic organ, may be accepted spontaneously following transplantation in experimental animals. In humans, it may be possible to withdraw immunosuppression in carefully selected stable patients without rejection and liver grafts may also confer protection on other grafts from the same donor (strain). Current information on liver allograft acceptance suggests hepatic resident immune cells (DC, T cells, KC, and potentially NK cells) cross-talk with parenchymal LSEC and hepatocytes, in conjunction with specific anti-inflammatory cytokines and signaling molecules to create a tolerogenic microenvironment. The phenomena of infiltrating T cell apoptosis in liver transplant recipients may be crucial to operational allograft tolerance, but underlying mechanisms are not well-understood. Recent findings reveal that MSC, especially liver graft-derived MSC, can suppress T cell-based immune responses. Fundamental differences in immune cell number, subset proportions, and responsiveness to tolerogenic cues may offer some explanation as to why liver allografts, but not other solid organ transplants, are readily accepted, and deserve further investigation.

Currently, non-invasive biomarkers to predict liver graft tolerance or rejection are promising. However, there are no definitive diagnostic criteria that have been widely validated and approved. Cutting-edge technologies, such as scRNAseq, provide a potential novel approach to predict liver transplant tolerance vs. rejection in the future. However, until accurate and non-invasive biomarkers are available, histopathological findings remain the gold standard to monitor the status of liver allografts.

To minimize side effects related to life-long immunosuppression, drug withdrawal has been advocated. Yet, drug withdrawal is not suitable for every patient. The development of novel cellular therapeutics, including MSC and regulatory cell therapy, is currently under evaluation in multiple trials worldwide to establish feasibility, safety, and efficacy. However, there are significant limitations to this approach, including cost, low cell yield, unpredictable function *in vivo*, and the dependence on the immunological status of each recipient. A combinatorial approach of CRISP/Cas9, chimeric Ag-receptor or gene-edited cellular therapy, combined with immunosuppression minimization is a possible strategy to promote clinical liver transplant tolerance, but will require the presence of adequate monitoring tools.

## Author Contributions

HD wrote the manuscript and designed the figure. YZ generated the tables. AT designed the outline of the manuscript and revised the manuscript. NR wrote and revised the manuscript. All authors have contributed to the editing of the manuscript.

## Conflict of Interest

The authors declare that the research was conducted in the absence of any commercial or financial relationships that could be construed as a potential conflict of interest.
